# Descriptive analysis of postmarket surveillance data for hip implants

**DOI:** 10.1002/pds.4971

**Published:** 2020-03-03

**Authors:** Josep Pane, Katia M. C. Verhamme, Irene Rebollo, Miriam C. J. M. Sturkenboom

**Affiliations:** ^1^ Department of Medical Informatics Erasmus Medical Center, University Medical Center Rotterdam Rotterdam Netherlands; ^2^ Eu2P European Programme in Pharmacovigilance and Pharmacoepidemiology University of Bordeaux Segalen Bordeaux France; ^3^ Julius Global Health University Medical Center Utrecht Utrecht Netherlands

**Keywords:** complaint data analysis, complaint data collection, medical devices, postmarket surveillance, spontaneous reports

## Abstract

**Purpose:**

Recent safety issues involving medical devices have highlighted the need for better postmarket surveillance (PMS) evaluation. This article aims to describe and to assess the quality of the PMS data for a medical device and, finally, to provide recommendations to improve the data gathering process.

**Methods:**

A descriptive analysis of medical device reports (MDRs) on the use of MRA, a specific type of hip implant replacement submitted to the Food and Drug Administration Manufacturer and User Facility Device Experience database from 1 January 2008 to 31 December 2017. The number of reports was described as the number of MDRs per unique MDR number and stratified by different variables. The quality was assessed by the level of completeness of the collected PMS data.

**Results:**

The total number of reports related to MRA was 2377, and the number of MDRs per year ranged between 84 in 2009 and 452 in 2017. Most of the reports were reported by manufacturer Depuy Johnson & Johnson and were reported by a physician. In 44.9% of the reports, the device problem was reported as “Unknown.” When the device problem was known, in the majority of cases, it was related to an implant fracture. The quality of the collected data was assessed as low due to missing information.

**Conclusion:**

The underlying data should meet high quality standards to generate more evidence and to ensure a timely signal generation. This case study shows that the completeness and quality of the MDRs can be improved. The authors propose the development of tools to ensure a more dynamic complaint data collection to contribute to this enhancement.

**KEY POINTS**

- The completeness and the quality of the data included in the medical device reports can be improved.
- New standards and safety tools should be developed to ensure a more dynamic complaint data collection process.

## 1 INTRODUCTION

An implantable medical device is a device that is partly or totally inserted into the human body or a natural orifice or is used to replace the surface of the body and is expected to stay in use for 30 days or more. Examples of implantable medical devices include dental implants, breast implants, hip implants and intraocular lenses. Surgical or medical procedures are used to insert, apply and remove implantable medical devices. To be classified as a non‐active implantable medical device (NAIMD), the medical device should not have an integral power source; all devices with a power source are considered active implantable medical devices (eg, pacemaker, cochlear implants…).^1^

Recent safety issues involving NAIMD have highlighted the need for better premarketing and postmarketing evaluation.^2^, ^3^ In the metal‐on‐metal (MoM) hip safety issue, thousands of patients around the world may have been exposed to high levels of toxic metals from failing hip implants. The chromium and cobalt ions from the MoM hip implants could enter into the tissues of patients with this type of hip implants, leading to reactions that damaged the muscle and bone, and led to revision procedures or left some patients with long‐term disability.^4^, ^5^, ^6^, ^7^ This safety issue was only identified by the Australian Health Authorities upon review of the Australian Orthopaedic Association National Joint Replacement Registry, and this finding was confirmed by the National Joint Replacement Registry of England, Wales, and Northern Ireland and the New Zealand Registry. This resulted in a worldwide recall of the MoM hip implants. The safety issue was highly publicized as MoM hip implants were approved for market use although lacking data derived from clinical trials. In addition, the manufacturers did not effectively review post‐market clinical data (including device registries containing postmarket surveillance information) and thus failed to identify and report this risk to the health authorities.^8^

A prior safety issue with Poly Implant Prothesis breast implant scandal^3^ had also contributed to the emerging growing demand to improve the current passive‐reactive postmarket surveillance (PMS) system of medical devices. An important part of this PMS system is the data collection of case (complaint) reports. To enhance the current surveillance system, it is important to measure and assess the quantity and quality of PMS data on medical devices.

Hip implants are NAIMD that are implanted during hip replacement surgery. Hip replacement surgery can be performed traditionally or by means of a minimally invasive technique. The main difference between the two procedures is the size of the incision and the type of prosthetic implant, either a total hip replacement or an MoM hip replacement.^9^ With approximately 1.4 million hip implant surgeries performed every year around the world, it is the most common joint replacement procedure. In the United States, over 231 000 surgeries are performed annually.^10^

Given the large use of hip implants and the need to improve medical device vigilance, we performed a case study and conducted a descriptive analysis of the PMS data from one of the most important publicly available spontaneous reports database,^11^ the Food and Drug Administration's (FDA) Manufacturer and User Facility Device Experience (MAUDE) database, to assess the quality and the quantity of these spontaneous reports using hip implants as a proof of concept, but our aim was not to investigate and compare the safety of individual (or specific) implants.

## 2 METHODS

### 2.1 Data Source

The PMS data for hip implants were extracted from the FDA MAUDE database. Medical device reports (MDRs) on the use of hip implant replacement were extracted from the FDA MAUDE of MDRs received by the FDA between 1 January 2008 and 31 December 2017. MAUDE contains MDRs received by the FDA on worldwide complaint data. Adverse events or technical complaint information of medical devices can be reported to the FDA via user facility (hospital), consumer or manufacturer.

Manufacturers must submit MDRs to the FDA “*when they become aware of information that reasonably suggests that one of their marketed devices may have caused or contributed to a death or serious injury or has malfunctioned and the malfunction of the device or a similar device that they market would be likely to cause or contribute to a death or serious injury if the malfunction were to recur. Manufacturers must send MDRs of such deaths, serious injuries and malfunctions to the FDA*”^12^ when they become aware of any of the events described above reported from any country in the world. The definition of serious injury is described below.

The FDA provides access to MAUDE information through three different tools: (a) an online simple (single‐parameter) interface, (b) advanced (multiparameter) search interface or (c) downloadable data files. These online search engines are extremely convenient; however, information obtained using these interfaces has some restrictions.^12^, ^13^, ^14^ In our study, we used both the online search interface and the downloadable datasets.

### 2.2 Outcomes

For this study, we were interested in reports related to the use of another type of hip implant (different from the MoM implant): the hip joint metal/ceramic/ceramic/metal semiconstrained cemented or uncemented prosthesis (FDA product code: MRA).^15^ We considered all events related to this type of device as events of interest. The FDA has a standardized vocabulary for adverse events and product problems. A total of 167 different event codes related to the use of the hip implant of interest (MRA) were analyzed. Malfunctions and serious injuries were classified according to the FDA regulatory definitions^16^; a serious injury is an injury or illness that is life‐threatening, results in permanent impairment/damage or necessitates medical/surgical intervention to preclude permanent impairment/damage. A malfunction stands for the failure of a device to meet its performance specifications or to perform as intended [Performance specifications include all claims made in the labeling of the device. The intended performance of a device refers to the intended use for which the device is labeled or marketed^16^].

The type of reporter was classified as unknown or known (physician, nurse, patient, pharmacist, administrative and known others). The type of reported adverse events was classified as malfunction and/or serious injury (for definitions, see above). The complaint sample availability and the corrective/remedial actions field were classified as Yes, No or NA.

### 2.3 Data management and analysis

The study period comprised 10 years, and data for this period (1 January 2008 through 31 December 2017) were obtained from three MAUDE downloadable datasets: the MDR Freedom of Information (FOI) master dataset, the Device Data dataset and the FOI Device Problem dataset. The advanced search interface dataset was used to obtain the name of all MRA hip implant manufacturers (filtering by date on which the report was received by the FDA (1 January 2008 through 31 December 2017) and product code MRA) with reported MDRs. The advanced search interface dataset was used to obtain the name of all MRA hip implant manufacturers. We had to standardize the manufacturer names by classifying each of the names from the manufacturer's column into eight different categories: Depuy Johnson & Johnson, Stryker, Wright Medical Technology, Zimmer, Encore, Stelkast, Exactech and Smith & Nephew.

We obtained the following information from each of the downloadable datasets:

- The MDR FOI Master dataset, filtering by the “manufacturer name” field for all the MRA Hip Implant Manufacturers available. The following variables were used: MDR report key, manufacturer name, type of event, report source, source type (country of origin; United States or foreign), reporter occupation, remedial actions and recalls.
- The Device Data dataset, filtering by “MDR report key.” The following variables were used from this dataset: MDR report key number (to link), device availability and device evaluated by the manufacturer.
- The FOI Device Problem dataset, filtering by “MDR report key.” The following information was used from this dataset: MDR report key Device Problem codes.

From these three datasets, one unique dataset was built using the “MDR report key,” which was available in the three downloadable datasets.

The data were analyzed using descriptive statistics, reporting counts, proportions and stratifications. Absolute numbers and percentages were described by manufacturer, brand name, type of event (death, injury, malfunction, NA, other), reporter's occupation, type of reported adverse events and product problems, complaint sample availability (whether the device is available for further investigation) and corrective/remedial actions. The numerator was the number of reports with MRA hip implants for a specific brand name, and the denominator was the total number of reports for MRA hip implants during the study period.

## 3 RESULTS

Eight MRA hip implant manufacturers reported MDRs to the FDA: Depuy Johnson & Johnson, Stryker, Wright Medical Technology, Zimmer, Encore, Stelkast, Exactech and Smith & Nephew. A total of 2377 unique FDA‐reportable complaints for MRA hip implants were received by the FDA from the manufacturer between 1 January 2008 and 31 December 2017, mostly originating in the United States (1807 reports, 76.0%). There was a high percentage of missing information. The proportion of reports with information on the type of device problem was 55.1% (in 44.9% of the reports, the device problem was reported as “Unknown”). The most frequently reported device problems included “implant fracture” (39.57%, 518 reports), “dislocation” (11.38%, 149 reports), “loss of osseointegration” (8.40%, 110 reports), “component/fitting issue” (2.60%, 34 reports),” material corrosion” (1.91%, 25 reports) and “metal shedding debris” (0.61%, 8 reports) (Table 1).

**Table 1:** Overview of the characteristics of medical device reporting (MDR) data reported between 1 January 2008 and 31 December 2017

Manufacturer	Number of MDRs (% of total)
Depuy Johnson & Johnson	1528 (64.28)
Stryker	546 (22.97)
Wright Medical Technology	192 (8.08)
Smith & Nephew	46 (1.94)
Zimmer	42 (1.77)
Exactech	12 (0.51)
Encore Medical	8 (0.34)
Stelkast	3 (0.13)
**Device Problem**	
Known	1309 (55.07)
Unknown	1068 (44.93)
**Known Device Problem**	n = 1309
Fracture/break/crack/scratched material	518 (39.57)
Dislodged/dislocated/displaced/disassembly/malposition/migration or expulsion of device	149 (11.38)
Loss of osseointegration/failure to bond	110 (8.40)
Component issue/connection issue/implant loose fitting issues/inadequacy of device shape/size/	34 (2.60)
Material corrosion/degradation/integrity/deformation/naturally worn	25 (1.91)
Metal shedding debris	8 (0.61)
Other	465 (35.52)
**Type of Event**	
Death	2 (0.08)
Serious injury	1714 (72.11)
Malfunction (no serious injury)	653 (27.47)
Other	4 (0.17)
Unknown	4 (0.17)
**Country of Origin**	
United States	1807 (76.02)
Foreign (rest of the world excluding United States)	514 (21.62)
Unknown	56 (2.36)
**Report Source Code**	
Manufacturer	2377
User facility	0
Distributor	0
Voluntary	0
**Reporter Occupation**	
Physician	913 (38.41)
Health professional other than physician	445 (18.72)
Attorney	89 (3.75)
Patient	28 (1.94)
Risk manager	14 (1.77)
Pharmacist	7 (0.30)
Company technician/representative	7 (0.30)
Others	752 (31.64)
Unknown	122 (5.13)
**Device Availability**	
Yes	627 (26.38)
No	1716(77.19)
Unknown	34 (1.43)
**Device Evaluated by Manucturer** a **(out of the available devices)**	
Yes	423 (67.46)
No	88 (14.04)
Unknown	116 (18.50)
**Remedial Action**	
Other	630 (39.12)
Recall	3 (0.12)
Modification/adjustment	1 (0.04)
Blank	1743 (73.33)
**Recalls‐Removal Correction Number**	
Z‐1749/1816‐2011 (Depuy Johnson & Johnson)	2 (66.67)
Blank (Depuy Johnson & Johnson)	1 (33.33)

a The device can only be evaluated by the manufacturer if it is available.

**Figure 1:**
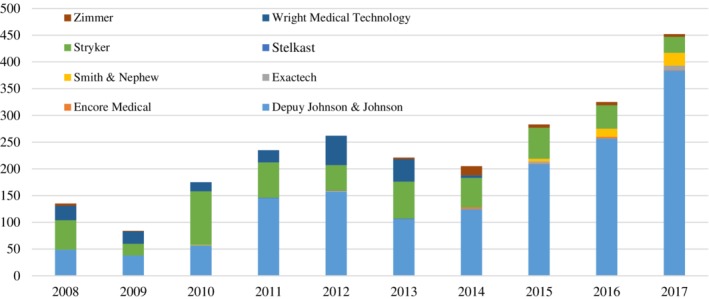
Number of medical device reports related to MRA hip implant by manufacturer per year from 1 January 2008 to 31 December 2017

Compared to all other MRA hip implant manufacturers, Depuy Johnson & Johnson had the most MDRs (64.28%, 1528 reports). For the other manufacturers, the number of reports for MRA hip implants were as follows: Stryker (22.97%, 546 reports), Wright Medical Tech (8.08%, 192 reports), Smith & Nephew (1.94%, 46 reports), Zimmer (1.77%, 42 reports), Exactech (0.51%, 12 reports), Encore Medical (0.34%, 8 reports) and Stelkast (0.13%, 3 reports). Death occurred in 0.08% (2 reports), and serious injury occurred in 72.11% (1714 reports) (Table 1). The number of yearly MDRs increased from 84 in 2009 to 452 in 2017 (Figure 1).

The reporter's occupation was reported in 94.9% of all reports, of which 40.5% of reporters were physicians (Table 1); 100% of the reports were submitted by the manufacturer, and no reports were submitted directly to the FDA by physicians, nurses, other healthcare providers or patients.

**Table 2:** Recommendations to improve Medical Device Reporting (MDR) and the Manufacturer and User Facility Device Experience (MAUDE) database

	Limitation	Recommendation	Owner
Event coding	Impossibility to identify patient harms and root causes associated with specific FDA device problem codes.	FDA patient codes and FDA investigational codes (methods, results and conclusions) publicly available in MAUDE.	FDA
Patient exposure	Lack of information about frequency of device use does not allow to estimate patient exposure.	The manufacturers could make their distribution/sales data available to the Health Authorities, upon request.	Global Health Authorities (including FDA), Manufacturers
Root cause identification	For the majority of reports the suspect sample is not available and cannot be evaluated by the manufacturer. Identifying the root cause of the event is especially difficult if the device in question has not been identified and directly evaluated by the manufacturer.	Global adoption of UDI: in order to identify the device and link the device to a serial number, UDI needs to be present on the device and readily accessible in the medical record. More guidance and training for the healthcare professionals on the importance of sending the device with all the adequate information (including UDI) to the manufacturer for evaluation, if the suspect device is explanted.	Reporting facilities, FDA, Manufacturers
Timely reporting	The MAUDE advanced search interface is updated monthly, and the search page reveals the date of the latest update. The FDA tries to include all reports received before the update, but the inclusion of some reports may be delayed.	More guidance and training on the importance of timely reporting should be provided to the different stakeholders involved in the complaint handling process.	Reporting facilities
Report source	Most of the MDRs from MAUDE come from spontaneous reports received from the manufacturer. This type of report may be associated with reporting bias.	Healthcare provider reports directly to the FDA need to be strongly encouraged via training and regulatory guidelines. Enriching the FDA MAUDE PMS data with data from medical device registries. In order to be able to link the registry data with the manufacturer reports data, common standardized dataset including UDI should be created.	FDA
Scope	MAUDE only includes FDA‐reportable complaints. If the complaint does not meet the FDA reporting criteria, the complaint will not be in MAUDE. MAUDE only includes complaints associated with medical devices that are marketed in the United States. If the medical device is not marketed in the United States, the complaint will not be in MAUDE.	Exchange of PMS data (including FDA‐nonreportable complaints and trend reports for FDA‐nonreportable complaints) between different Health Authorities. Development of a global repository to store global PMS data for medical devices.	IMDRF, Global Health Authorities (including FDA)

Abbreviations: FDA, Food and Drug Administration; PMS, PostMarket Surveillance; UDI, Unique Device Identifier.

The proportion of reports with information on the suspect device availability was higher (98.57%, 2343 reports), and the device was only available in 26.38% of the reports (627 reports). The manufacturer was only able to evaluate the suspect sample in 17.80% of the reports (423 reports). Without a sample, it is more difficult to identify the root cause of the event and take appropriate actions. A remedial action was only identified for 26.67% (634 reports) of the reports, and only 0.47% (3 reports) of the remedial actions were associated with a recall. The three reports associated with a recall came from the same manufacturer, namely, Depuy Johnson & Johnson (Table 1).

## 4 DISCUSSION

This case study on medical device reporting on MRA hip implants to the FDA demonstrated some key findings. First, beyond the United States, very few reports were received from other countries, and no reports were submitted by physicians, nurses, other healthcare providers or patients. Second, most reports were on serious injury, and the most frequently reported device problem was “fracture of the hip implant.” Third, completeness of information in the reports was poor, and often, the suspect sample was not sent to the manufacturer and therefore could not be evaluated, which hampers the root cause analysis.

**Figure 2:**
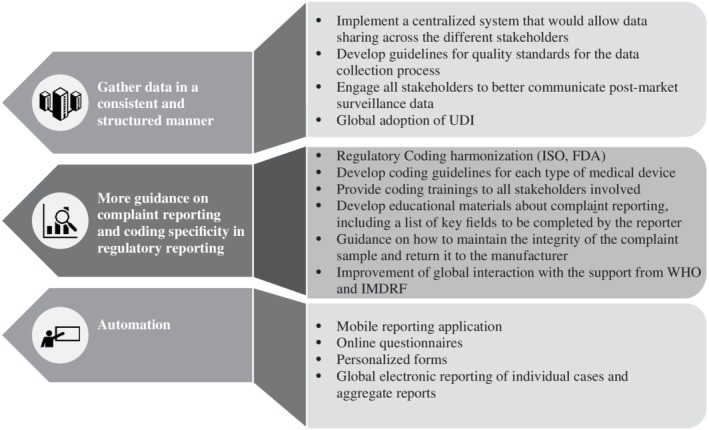
Recommendations to obtain better postmarket complaint data for medical devices. FDA, Food and Drug Administration; IMDRF, International Medical Device Regulators Forum; ISO, International Organization for Standardization; UDI, Unique Device Identifier; WHO, World Health Organization

These results underline the need to obtain better postmarket complaint data for medical devices within the United States and beyond (Figure 2). Improvements can be made in the reporting itself, the collecting database and the awareness of the different stakeholders involved in the safety evaluation process. High quality standards with a consistent and structured approach are needed to optimally gather MDR. More specificity in regulatory reporting and harmonized regulatory coding might help to generate better evidence to ensure an accurate and well‐timed signal generation.

To address this problem of quality issues in reporting, as well as in the completeness of data, the manufacturer and the health authorities should engage the reporter (patient or healthcare professional) in the complaint data collection process. In addition, the regulators and the manufacturers could provide tools to healthcare providers and users that would give more guidance on complaint reporting and appropriate coding. Examples of such tools could be the development of educational material for the healthcare professionals about complaint reporting, including a list of key fields to be completed by the reporter; guidance providing instructions on how to manipulate complaint samples that have been in contact with human fluids and how to return them to the manufacturer, ensuring safe transport to maintain the integrity of the complaint sample; the regulatory coding harmonization and global implementation across jurisdictions; and coding guidelines developed by the regulators for each type of medical device and provided to all the stakeholders.

To stimulate reporting and facilitate timely reporting, the process should be automated, and healthcare professionals could be involved in the use of digital reporting tools such as mobile applications, online questionnaires, personalized forms and global electronic reporting of individual cases and aggregate reports, which can lead to quality improvement of the collected information.^17^ Table 2 provides recommendations not only on how to improve MDR reporting but also recommendations on how to improve data collection in the MAUDE database. In addition to the limitations described in Table 2, our results also have additional limitations as postmarketing complaint data is prone to reporting bias.^18^ Moreover, a descriptive analysis of postmarketing complaint data does not allow to control patient predisposing factors such as family history, health condition or previous surgeries. Therefore, we recommend enriching the FDA MAUDE data with PMS data from medical device registries. To link the registry data with the spontaneous report data from MAUDE, a common Unique Device Identifier (UDI) should be created. The UDI enables the unequivocal identification of the medical device by providing a single global identifier that can be used to link and integrate the existing FDA MAUDE database with medical device registries.^19^ The global use of a UDI facilitates traceability throughout distribution and allows the recording of medical devices used in patients. The UDI makes it possible to link patient, device and adverse event/product problem and/or related data repositories. This information can help the different stakeholders involved in the safety evaluation of medical devices to quickly gather and evaluate spontaneous reports or data from registries and act accordingly.

To improve the ability to signal problems on a global scale, a global harmonization and repository/database (similar to the World Health Organization [WHO] Vigibase for medicinal products) should be created to allow sharing of information across the different stakeholders (health authorities, users, manufacturers, notified bodies and health professionals) in addition to the development of quality standards for the data gathering and a global centralized database to collect and store reports related to medical devices. To ensure success, regulators should partner with the manufacturers, which could be facilitated by the improvement of worldwide interactions between different stakeholders with support from the WHO, International Council for Harmonization of Technical Requirements for Pharmaceuticals for Human Use (ICH), Council for International Organizations of Medical Sciences (CIOMS) and the International Medical Device Regulators Forum (IMDRF). WHO, ICH and CIOMS should provide their experience and lessons learned from the global harmonization of medicinal products, and IMDRF should play a significant role in the standardization of quality standards across the different regulatory bodies.^20^

In conclusion, there is an urgent need for better PMS for medical devices, which we demonstrate through the MRA hip implant example. The quality of postmarket complaint data and their timely collection are crucial for the validity of the complaint reports. It is time to face current challenges such as the lack of quality standards, lack of specificity in regulatory reporting, lack of harmonized coding and lack of engagement from reporters at the time to send samples back for analysis. We recommend that the different stakeholders in this process (manufacturers, health authorities, healthcare professionals and patients) work together to overcome these challenges.

## CONFLICT OF INTEREST

The authors declare no conflict of interest.
